# Association between polymorphisms of OGG1, EPHA2 and age-related cataract risk: a meta-analysis

**DOI:** 10.1186/s12886-016-0341-y

**Published:** 2016-09-29

**Authors:** Hongxu Zhang, Jianguang Zhong, Zhenyu Bian, Xiang Fang, You Peng, Yongping Hu

**Affiliations:** 1 Department of Ophtalmology, Hangzhou First People’s Hospital, Hangzhou Hospital Affiliated to Nanjing Medical University, Huansha Road No. 261, Hangzhou, 310006 Zhejiang People’s Republic of China; 2 Department of Orthopaedics, Hangzhou First People’s Hospital, Hangzhou Hospital Affiliated to Nanjing Medical University, Hangzhou, 310006 Zhejiang People’s Republic of China; 3 Department of Central Laboratory, Hangzhou First People’s Hospital, Hangzhou Hospital Affiliated to Nanjing Medical University, Hangzhou, 310006 Zhejiang People’s Republic of China; 4 Department of Surgical Oncology, Hangzhou First People’s Hospital, Hangzhou Hospital Affiliated to Nanjing Medical University, Hangzhou, 310006 Zhejiang People’s Republic of China

**Keywords:** Age-related cataract, OGG1, EPHA2, Polymorphism, Meta-analysis

## Abstract

**Background:**

Evidences have identified the correlation of 8-oxoguanine DNA glycosylase-1 (OGG1) and eph-receptor tyrosine kinase-type A2 (EPHA2) polymorphisms in age-related cataract (ARC) risk. However, the results were not consistent. The objective of this study was to examine the role of these two gene polymorphisms in ARC susceptibility.

**Methods:**

Eligible case–control studies published between January 2000 and 2015 were searched and retrieved in the electronic databases. The odds ratio with 95 % confidence interval (CI) was employed to calculate the strength of the relationship.

**Results:**

We totally screened out six articles, including 5971 cataract patients and 4189 matched controls. Three variants were contained (OGG1 rs1052133; EPHA2 rs7543472 and rs11260867). For OGG1 rs1052133, we detected a significant correlation between OGG1 polymorphism and ARC risk under the heterogenous model (CG vs. CC: OR = 1.34, 95 % CI = 1.06–1.70, *P* = 0.01) and dominant model (GG+CG vs. CC: OR = 1.45, 95 % CI = 1.16–1.81, *P* = 0.001), especially in patients with cortical cataract of subgroup analysis by phenotypes (*P* < 0.05). For EPHA2 rs7543472 and rs11260867, we did not find a positive association between these two mutations and ARC susceptibility in total cases. Subgroup analysis by phenotypes of cataract showed that only in cortical cataract, genotypes of rs7543472 under the allele model, homogenous model and recessive model; genotypes of rs11260867 under the heterogenous model and dominant model were associated with ARC risk.

**Conclusions:**

OGG1 rs1052133 (CG and CG+GG genotypes) might be risk factor for ARC, particularly in cortical cataract risk. EPHA2 rs7543472 (T allele and TT genotype) and rs11260867 (CG and GG+CG genotypes) might be associated with cortical cataract.

## Background

Cataract is a leading cause of blindness and visual impairment throughout the world, increasing the public health and economic burden of this disease [1]. According to the Global estimates of visual impairment, approximate 51 % of blindness and 33 % of visual impairment were estimated due to cataract between 2000 and 2010 [2]. In addition, there are racial/ethnic disparities in the prevalence of cataract [3]: Cataract of Europeans accounts for 50 % (WHO criteria) to 65 % (US criteria) of unilateral visual impairment, and 45 % (US criteria) of 5-year incident bilateral visual impairment [4]; Asian populations had a higher prevalence and earlier age of onset of cataract than Europeans [5]; while the prevalence of cataract was lower in Africans compared with Europeans [6]. The major risk factors for cataract are age, as well as several demographic and lifestyle characteristics [7, 8]. Approximately 80 % of cataract is age-related cataract (ARC) [9]. Based on the location of the opacity in the lens, ARC is classified as cortical cataract, nuclear cataract, or posterior subcapsular cataract [10]. The prevalence of ARC is rising, with a prediction that 30.1 million Americans will be affected by 2020 [11]. However, its etiology is multi-factorial and not fully understood to date.

Etiological research have found that the set of genes were associated with cataract, especially for ARC [12]. In recent years, 8-oxoguanine DNA glycosylase-1 (OGG1) and eph-receptor tyrosine kinase-type A2 (EPHA2) have been identified as key regulators in lens clarity. OGG1 is located in human chromosome 3p26.2, and its protein is a key enzyme in base excision repair (BER) pathway [13]. It is involved in maintaining genome integrity and preventing cancer development [14]. OGG1 could be used as a therapeutic target for certain types of cancer in monotherapy or combination therapy [15]. OGG1 is also implicated in oxidative mechanisms which play an important role in the pathogenesis of ARC, and might increase the risk of developing ARC [16]. Single nucleotide polymorphism (SNP) of OGG1 was shown to be a risk factor for oxidative pathologies. The most studied variant was OGG1 gene rs1052133 (Ser326Cys), a C to G transversion at nucleotide 1245, leading to a serine to cysteine substitution at residue 326 located in the C-terminal domain of the protein. The Cys326 protein has been shown to have about 7 times weaker 8-hydroxyguanine-repair capacity than Ser326 protein [17]. Homozygous carriers of the S326C OGG1 polymorphism presented reduced repair activity, and C326 OGG1 homozygous carriers might be at increased risk of oxidative pathologies [18].

EPHA2, located in human chromosome 1p36, is a member of the Eph receptor tyrosine kinases family [19]. It is an important regulator of tumor initiation, neo-vascularization and metastasis in a wide range of epithelial and mesenchymal cancers [20, 21]. EPHA2 is highly expressed in aggressive human cancers, and also offers opportunities for Eph/ephrin-based targeted drug delivery and imaging [22, 23]. EPHA2 protein is expressed in human and mouse lens [24, 25]. Multiple mutations in the EPHA2 gene have been recently shown to cause cataracts in humans, contributing to the destabilization of the receptor and the loss of cell migration activity [26]. The TT genotype of rs7543472 was shown to be associated with ~2× increased risk for cataracts; rs3754334 might be a variant on the EPHA2 gene that is commonly associated with the risk for ARC in different ethnical and geographical populations [27].

Although epidemiologic studies have identified the correlation of these genes polymorphisms in ARC risk, however, the results remain inconclusive. Therefore, we conducted this meta-analysis to establish the true association between OGG1 and EPHA2 SNPs and the risk for ARC.

## Methods

### Identification of eligible articles

We performed a comprehensive literature search in the following electronic databases of Medline, PubMed, Springer and Elsevier to retrieve relevant articles published between January 2000 and 2015. The key terms were “cataract” or “age-related cataract”, “8-oxoguanine glycosylase-1 or OGG1 or DNA repair gene”, “Eph-receptor tyrosinekinase-type A2 or EPHA2”, and “variant or polymorphism” as well as their combinations. References of related studies were manually searched to obtain more sources. Only studies written in English were included in this meta-analysis.

### Criteria for inclusion and exclusion

The inclusion criteria were as follows: 1) case–control studies concerning the role of OGG1 or EPHA2 polymorphisms in ARC risk; 2) patients with ARC was defined as lens opacity along with disturbance of vision and were over 50 years old (cataract status was determined by lens examination using a slit-lamp biomicroscope; lens opacities were determined using the Lens Opacities Classification System III [28]); controls were age-, sex-, and ethnically matched individuals without history of cataract, hypertension, or other ocular diseases; 3) the genotype information were available to extract, and the results were expressed as odds ratio (OR) with 95 % confidence interval (CI); 4) when the same authors or laboratories reported the issue among the same populations in more articles, only the recent full-text articles were included; and 5) genotype distribution of control for a certain polymorphism must be in Hardy-Weinberg equilibrium (HWE).

The exclusion criteria were: 1) review reports or conference papers; 2) without control group; 3) studies with duplicate data; and 4) genotype information couldn’t be extracted.

### Quality assessment and data extraction

Two investigators independently assessed the quality of related articles. Any disagreement was subsequently resolved by discussion with another expert to reach a consensus on all of the items. The following information was extracted from each article: first author, year of publication, country, ethnicity, mean age, sample size, genotyping method, and genotype distribution in cases and controls.

### Statistical analysis

The strength of association between polymorphisms of OGG1, EPHA2 and ARC risk was measured by the pooled ORs with its 95 % CI. The Z test was employed to determine the significance of the pooled ORs, and a *P* value less than 0.05 was considered statistically significant. For all the genetic polymorphisms, the comparison models (allelic model; homogenous model; heterogenous model; dominant model; and recessive model) were examined. The I^2^ test and the Q-statistic test were used to estimate between-study heterogeneity. The random-effect model was employed when the *P*-value less than 0.10 for the Q-test and I^2^ more than 50 %; otherwise, the fixed-effects model was used. The evidence of publication bias was assessed by visual funnel plot inspection. Statistical analyses were conducted in Review Manager (RevMan version 5.3, the Cochrane Collaboration, Oxford, England). All the tests were two-sided.

## Results

### Literature search and meta-analysis databases

**Fig. 1:**
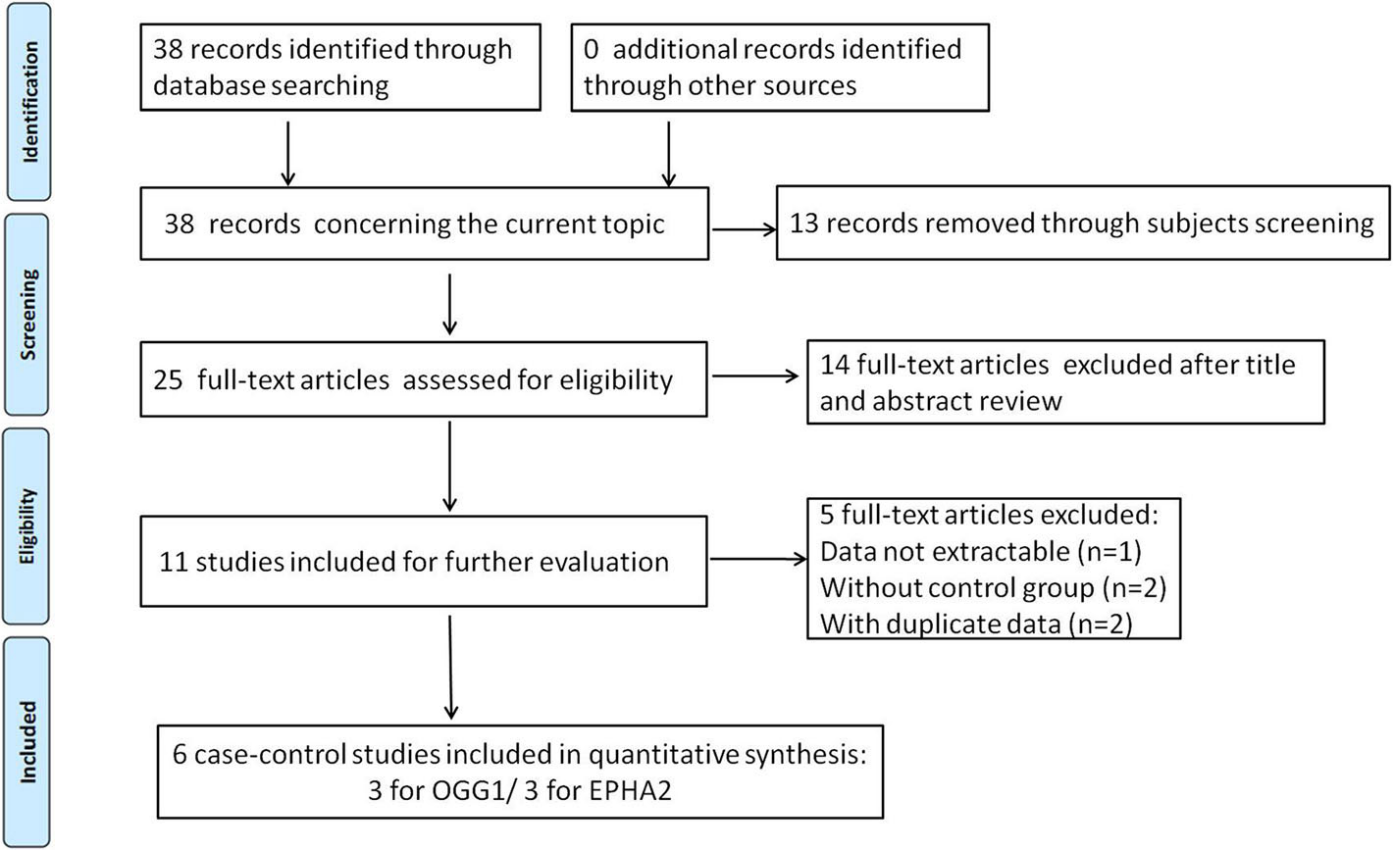
Flow chart of selection process

**Fig. 2:**
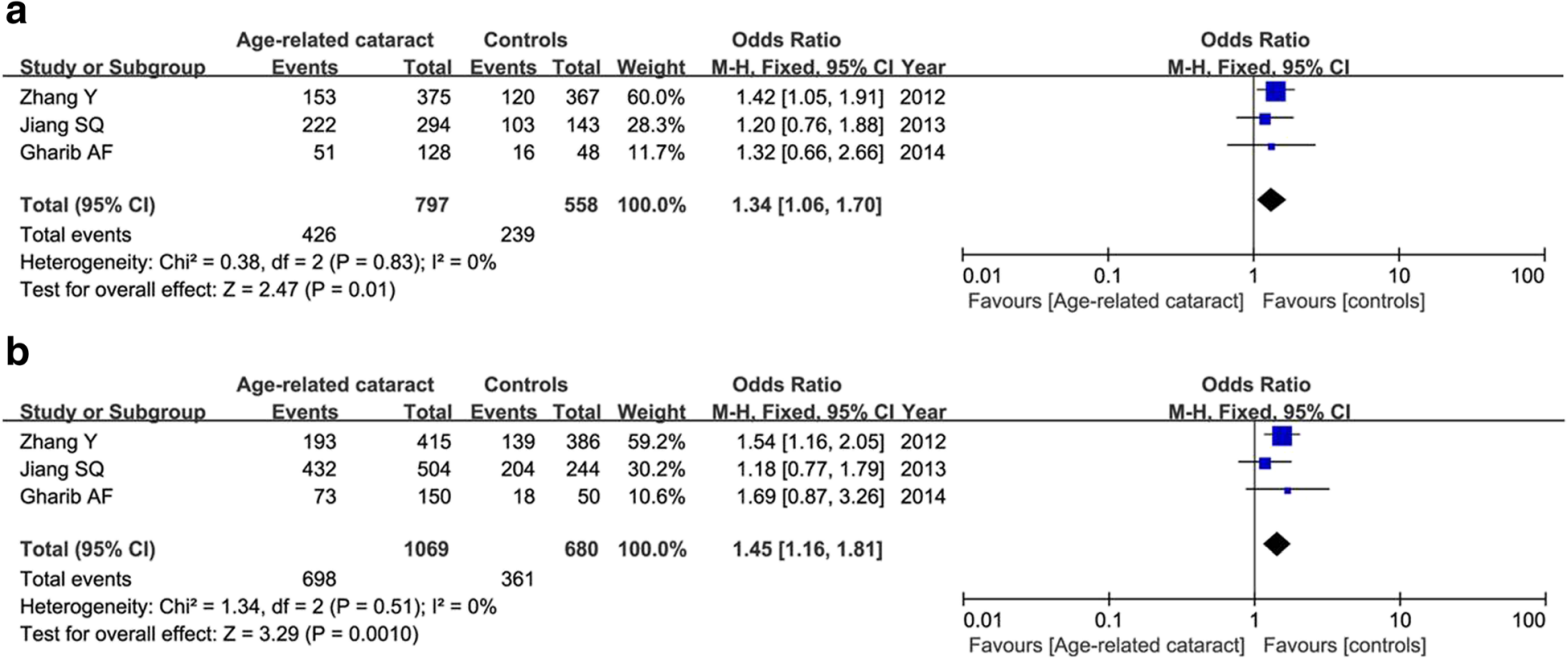
Meta-analysis of the relationship between the OGG1 rs1052133 and age-related cataract under the heterogenous model (**a**: CG vs. CC) and dominant model (**b**: GG+CG vs. CC)

We finally screened out 6 relevant articles, including 5971 cataract patients and 4189 controls. Figure 1 presented the flow diagram of searching process. The six studies were conducted in five countries (USA [29], India [30], China [31, 32], Sweden [33], Egypt [34]) and contained three SNPs (one for OGG1: rs1052133; two for EPHA2: rs7543472 and rs11260867). All these articles were written in English, and genetic polymorphisms of OGG1 and EPHA2 were measured by polymerase chain reaction. The genotype distribution in controls were all in accord with HWE (*P* > 0.05). Tables 1 and 2 listed the essential information of included studies in this meta-analysis. Figure 2 presented the distribution of genotype information.

**Table 1:** Main characteristics of included studies in this meta-analysis

First author	Year	Country	Ethnicity	Mean age		Sample size		Genotype methods
				ARCs	Controls	ARCs	Controls	
Shiels A [29]	2008	USA	European	75.7 ± 7.9	74.5 ± 7.6	213	104	PCR
Sundaresan P [30]	2012	India	Asian	–	–	4198	3220	PCR
Zhang Y [31]	2012	China	Asian	67.17 ± 6.92	65.77 ± 6.49	415	386	PCR-RFLP
Jiang SQ [32]	2013	China	Asian	70.9 ± 8.2	60.2 ± 5.7	504	244	PCR
Celojevic D [33]	2014	Sweden	European	72 ± 8.7	66 ± 6.9	491	185	PCR
Gharib AF [34]	2014	Egypt	African	60.33 ± 6.22	67.83 ± 5.54	150	50	PCR

*ARC* age-related cataract, − not available, *PCR-RFLP* polymerase chain reaction-restriction fragment length polymorphism

**Table 2:** Genotype distribution of OGG1 and EPHA2 polymorphisms in cataract cases and controls

First author	ARCs					Controls					HWE
rs1052133	CC	CG	GG	C	G	CC	CG	GG	C	G	
Zhang Y [31]	222	153	40	597	233	247	120	19	614	158	0.68
Jiang SQ [32]	72	222	210	366	642	40	103	101	183	305	0.29
Gharib AF [34]	77	51	22	205	95	32	16	2	80	20	1.00
rs7543472	TT	TC	CC	T	C	TT	TC	CC	T	C	
Shiels A [29]	146	53	5	345	63	58	41	3	157	47	0.40
Sundaresan P [30]	202	1419	2569	1823	6557	128	1054	2028	1310	5110	0.83
Celojevic D [33]	298	163	30	759	223	115	58	12	288	82	0.46
rs11260867	CC	CG	GG	C	G	CC	CG	GG	C	G	
Shiels A [29]	4	43	166	51	375	1	33	68	35	169	0.38
Sundaresan P [30]	25	623	3527	673	7677	24	448	2725	496	5898	0.50
Celojevic D [33]	317	158	16	792	190	121	58	6	300	70	0.96

*ARC* age-related cataract, *HWE* Hardy-Weinberg Equilibrium

### Association between OGG1 rs1052133 (C/G) and ARC risk

Table 3 showed the results of test for relationship between OGG1 and EPHA2 polymorphisms and ARC risk based on different genetic models in total and subgroup analysis.

**Table 3:** Meta-analysis of OGG1 and EPHA2 polymorphisms in ARC based on different genetic models

Comparisons	Total		Cortical cataract		Nuclear cataract		PSC	
	OR (95 % CI)	*P*	OR (95 % CI)	*P*	OR (95 % CI)	*P*	OR (95 % CI)	*P*
rs1052133								
G vs. C	1.36 (0.99, 1.87)	0.05	1.50 (0.97, 2.32)	0.07	1.23 (0.98, 1.55)	0.07	1.26 (0.94, 1.68)	0.12
GG vs. CC	1.88 (0.96, 3.71)	0.07	2.18 (0.90, 5.28)	0.08	1.45 (0.87, 2.44)	0.16	1.61 (0.83, 3.09)	0.16
CG vs. CC	1.34 (1.06, 1.70)	0.01	1.43 (1.04, 1.96)	0.03	1.32 (0.93, 1.86)	0.12	1.22 (0.79, 1.88)	0.37
GG+CG vs. CC	1.45 (1.16, 1.81)	0.001	1.54 (1.15, 2.07)	0.004	1.37 (0.99, 1.90)	0.06	1.32 (0.88, 1.99)	0.18
GG vs. CG+CC	1.65 (0.82, 3.32)	0.16	1.83 (0.78, 4.29)	0.17	1.18 (0.79, 1.75)	0.43	1.30 (0.78, 2.17)	0.31
rs7543472								
T vs. C	1.13 (0.92, 1.38)	0.25	1.16 (1.01, 1.33)	0.03	1.04 (0.75, 1.43)	0.83		
TT vs. CC	1.23 (0.99, 1.52)	0.06	1.52 (1.05, 2.18)	0.03	1.08 (0.84, 1.39)	0.55		
TC vs. CC	1.06 (0.96, 1.17)	0.22	0.99 (0.82, 1.20)	0.94	1.03 (0.92, 1.15)	0.61		
TT+TC vs. CC	1.08 (0.99, 1.19)	0.10	1.06 (0.88, 1.27)	0.53	1.04 (0.93, 1.16)	0.49		
TT vs. TC+CC	1.24 (0.90, 1.72)	0.18	1.54 (1.18, 2.01)	0.001	1.11 (0.72, 1.70)	0.63		
rs11260867								
G vs. C	0.99 (0.89, 1.11)	0.88	0.89 (0.73, 1.07)	0.20	1.09 (0.84, 1.40)	0.52		
GG vs. CC	1.14 (0.71, 1.83)	0.59	0.62 (0.31, 1.24)	0.18	1.38 (0.76, 2.49)	0.29		
CG vs. CC	1.08 (0.80, 1.47)	0.60	0.66 (0.43, 1.00)	0.05	1.46 (0.95, 2.23)	0.08		
GG+CG vs. CC	1.08 (0.80, 1.45)	0.62	0.66 (0.44, 0.99)	0.04	1.43 (0.94, 2.18)	0.09		
GG vs. CG+CC	1.16 (0.74, 1.81)	0.52	1.19 (0.55, 2.59)	0.66	0.95 (0.83, 1.10)	0.50		

*PSC* posterior subcapsular cataract, *OR* odds ratio, *95 % CI* 95 % confidence interval

**Fig. 3:**
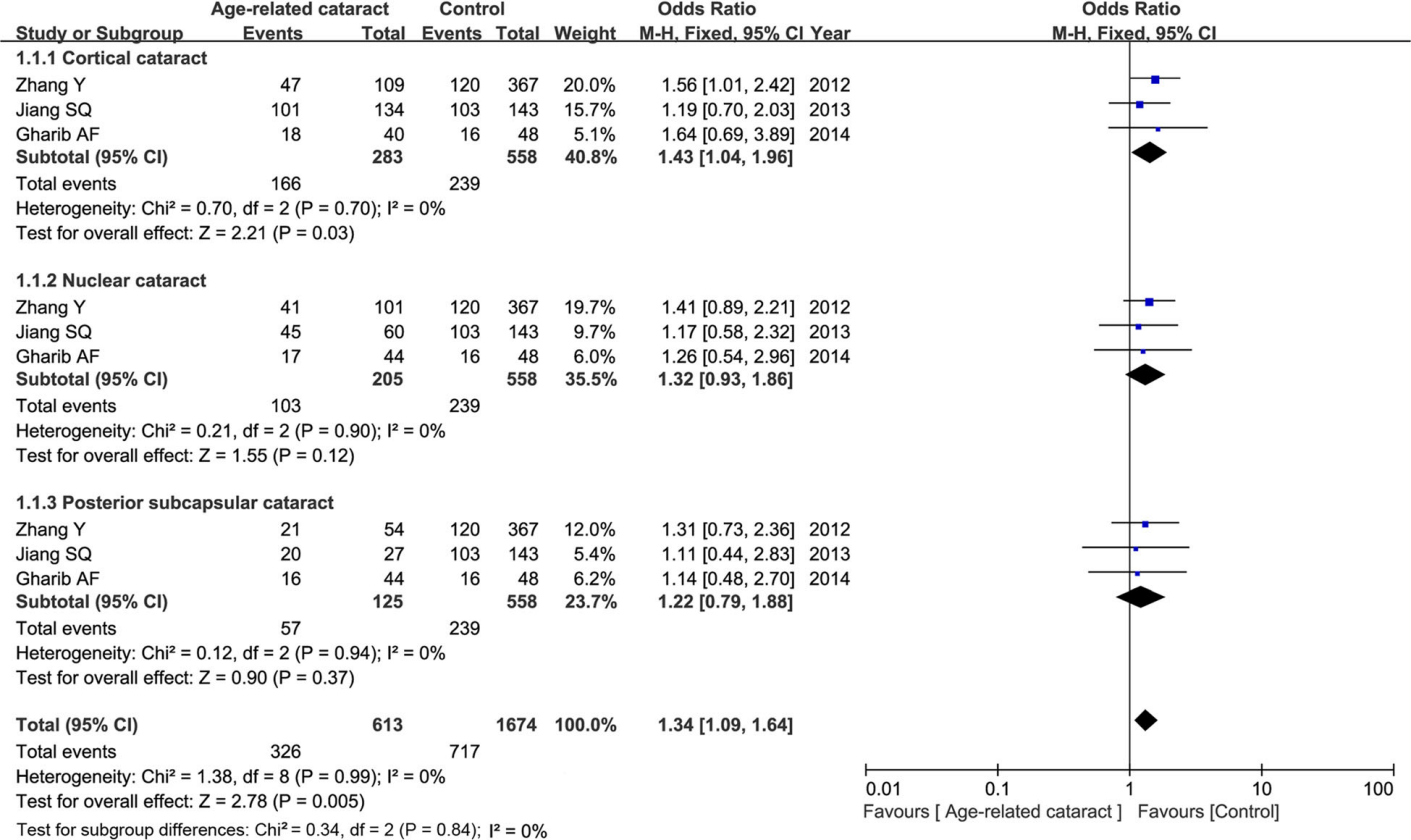
Forest plot of the relative strength of the association between OGG1 rs1052133 and different types of cataract under the heterogenous model (CG vs. CC)

Three articles concerned the OGG1 variant, including 1069 patients and 680 controls. Although the frequency of G allele (minor) was shown to be higher in ARC cases than that in controls (45.4 versus 35.5 %), our result found that the G allele was not associated with ARC susceptibility in the random-effect model (OR = 1.36, 95 % CI = 0.99–1.87, *P* = 0.05). This insignificance was also found under the homogenous model and recessive model (*P* > 0.05). But we detected a significant correlation between OGG1 polymorphism and ARC risk in the heterogenous model (CG vs. CC: OR = 1.34, 95 % CI = 1.06–1.70, *P* = 0.01) and dominant model (GG+CG vs. CC: OR = 1.45, 95 % CI = 1.16–1.81, *P* = 0.001) in the fixed-effect model as shown in Fig. 2. Subgroup analysis by phenotypes of cataract showed that only in cortical, not nuclear or posterior subcapsular cataract, OGG1 variant was associated with increased the risk of ARC under the heterogenous model (CG vs. CC: OR = 1.43, 95 % CI = 1.04–1.96, *P* = 0.03) and dominant model (GG+CG vs. CC: OR = 1.54, 95 % CI = 1.15–2.07, *P* = 0.004). Figure 3 showed the relative strength of the association between OGG1 rs1052133 and different types of cataract under the heterogenous model.

### Association between EPHA2 rs7543472 (C/T), rs11260867 (C/G) and ARC risk

**Fig. 4:**
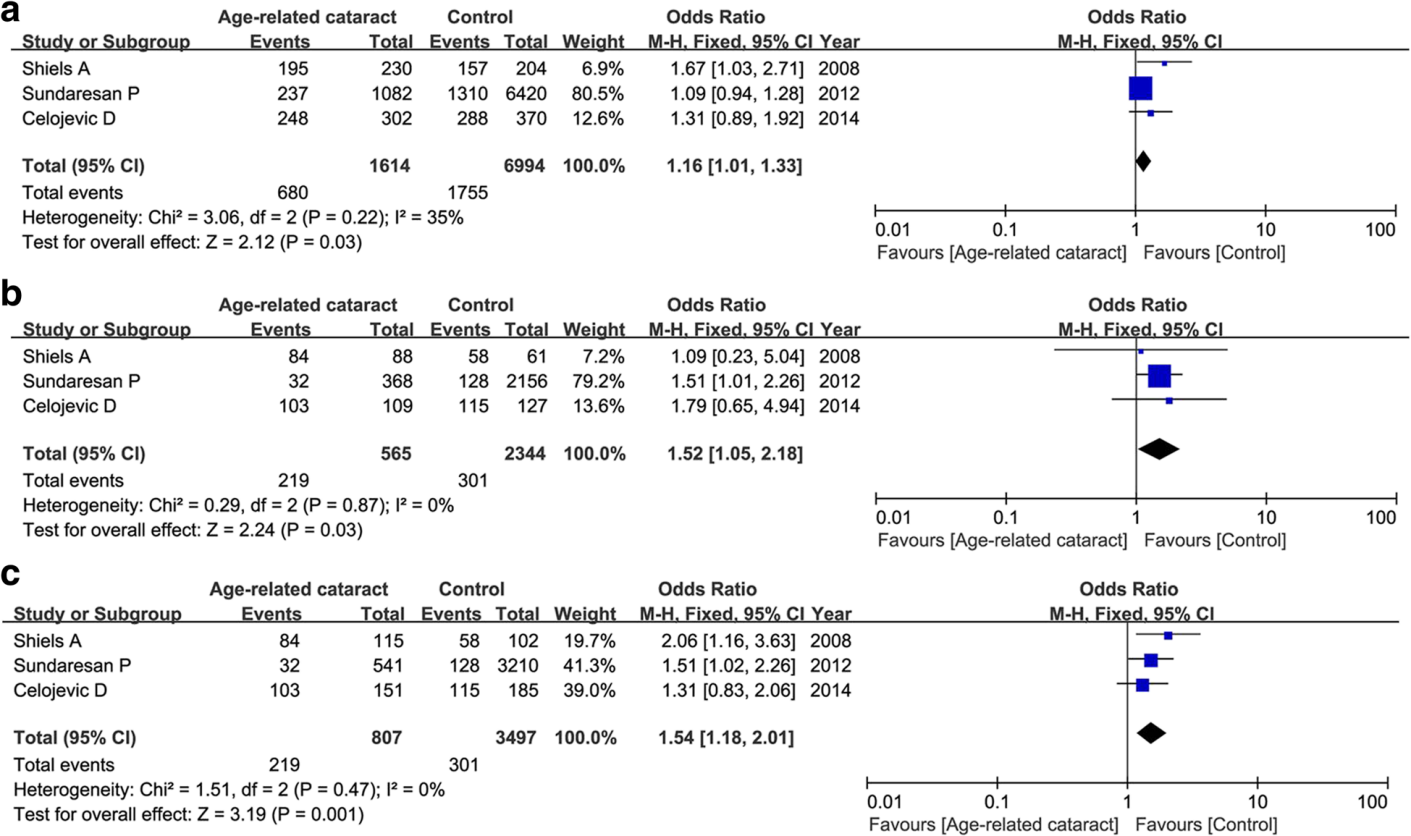
Meta-analysis of correlation of EPHA2 rs7543472 in cortical cataract under the allele model (**a**: T vs. C), homogenous model (**b**: TT vs. CC) and recessive model (**c**: TT vs. TC+CC)

Three articles contained 4902 cases and 3509 controls evaluating the association of EPHA2 genetic polymorphisms and ARC occurrence. For rs7543472, our result found that this variant was not associated with ARC risk under any genetic models (T vs. C: OR = 1.13, 95 % CI = 0.92–1.38, *P* = 0.25; TT vs. CC: OR = 1.23, 95 % CI = 0.99–1.52, *P* = 0.06; TC vs. CC: OR = 1.06, 95 % CI = 0.96–1.17, *P* = 0.22; TT+TC vs. CC: OR = 1.08, 95 % CI = 0.99–1.19, *P* = 0.10; TT vs. TC+CC: OR = 1.24, 95 % CI = 0.90–1.72, *P* = 0.18). Subgroup analysis by phenotypes of cataract showed a significant relationship between rs7543472 and cortical cataract under the allele model (OR = 1.16, 95 % CI = 1.01–1.33, *P* = 0.03), homogenous model (OR = 1.52, 95 % CI = 1.05–2.18, *P* = 0.03) and recessive model (OR = 1.54, 95 % CI = 1.18–2.01, *P* = 0.001) in the fixed-effect model as shown in Fig. 4. No association was detected between rs7543472 and patients with nuclear cataract under any genetic models.

**Fig. 5:**
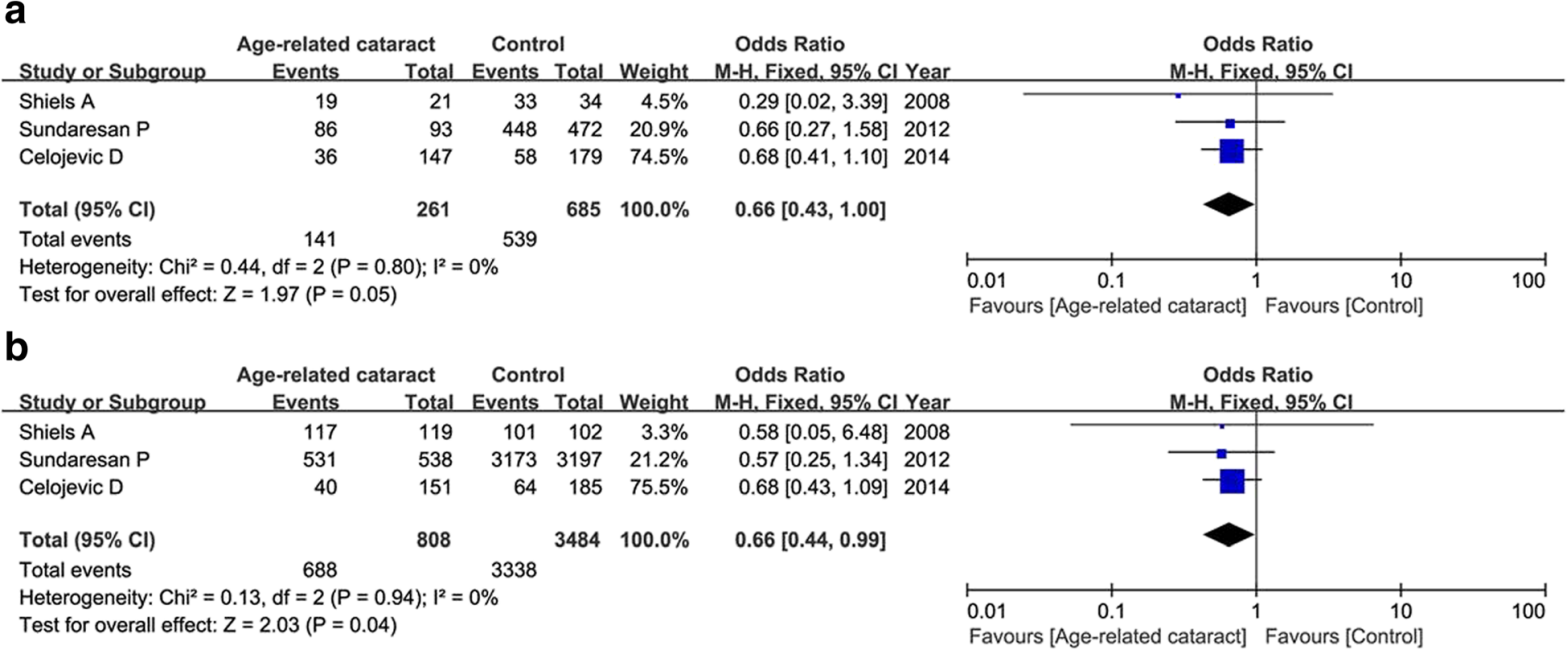
Forest plot of the association between EPHA2 rs11260867 and cortical cataract risk under the heterogenous model (**a**: CG vs. CC) and dominant model (**b**: GG+CG vs. CC)

For rs11260867, we did not observe a significant positive correlation between this variant and ARC risk under any genetic models as well (Table 3). Subgroup analysis by phenotypes of cataract showed that this variant was associated with increased the risk of cortical cataract, not nuclear cataract under the heterogenous model (CG vs. CC: OR = 0.66, 95 % CI = 0.43–1.00, *P* = 0.05) and dominant model (GG+CG vs. CC: OR = 0.66, 95 % CI = 0.44–0.99, *P* = 0.04) as shown in Fig. 5.

### Sensitivity analysis and publication bias evaluation

**Fig. 6:**
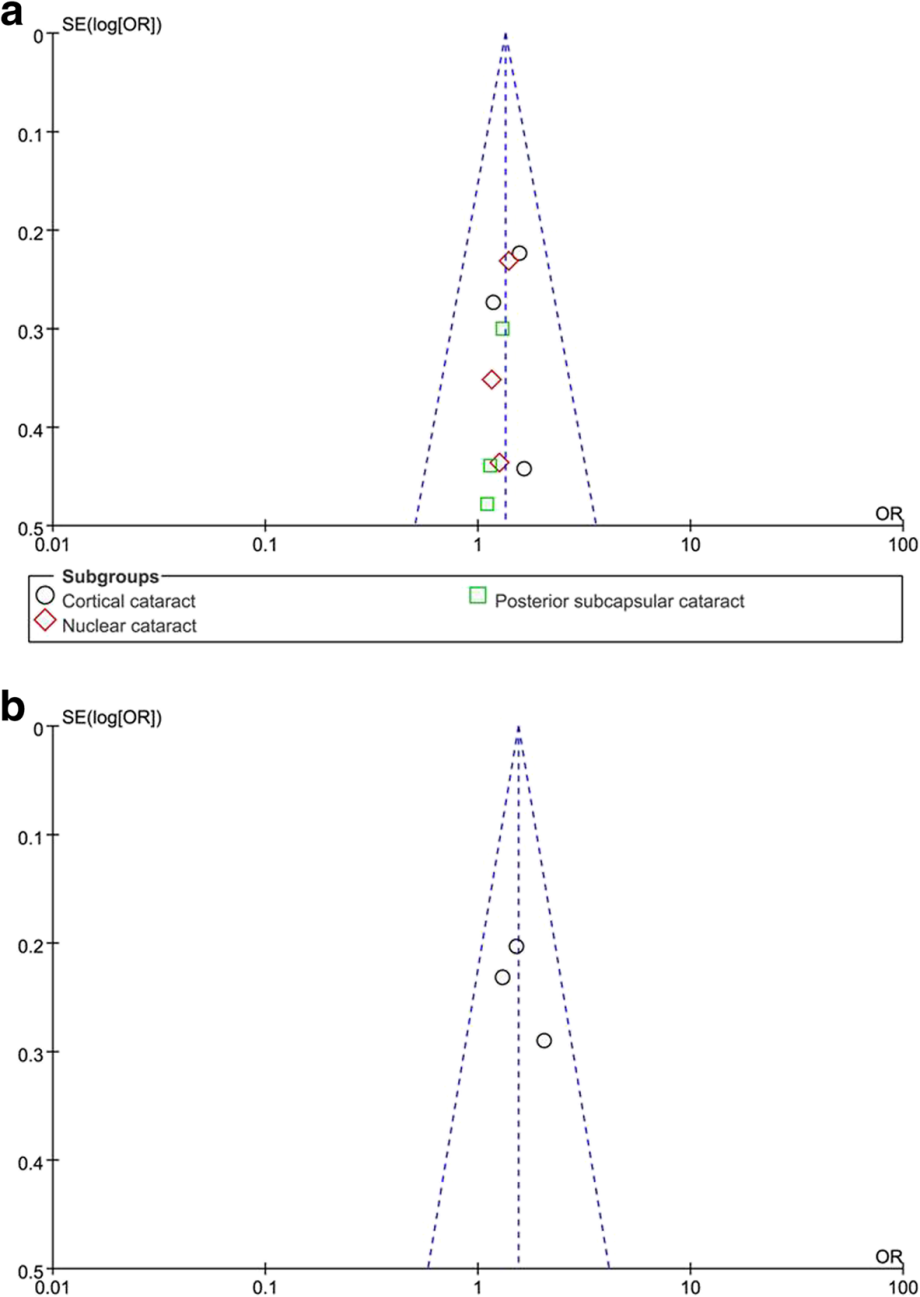
Funnel plot of **a**: OGG1 rs1052133 under the heterogenous model (CG vs. CC); **b**: EPHA2 rs7543472 under the recessive model (TT vs. TC+CC) in cataract risk

To confirm whether each included study influences the overall results or not, we successively omitted each single study, respectively. Our result found that the pooled ORs were not significantly changed. The funnel plots were used to evaluate the publication bias. All the plots were found to be roughly symmetrical, indicating no publication bias presented as shown in Fig. 6.

## Discussion

In this meta-analysis, we totally identified six articles concerning three genetic polymorphisms. Our results showed that CG genotype and GG+CG genotype of OGG1 rs1052133 were associated with increased the risk of ARC, in particular with cortical cataract. This significant relationship was not found in EPHA2 polymorphisms (rs7543472 and rs11260867), however, subgroup analysis by phenotypes of cataract showed that only in cortical cataract, the genotypes of rs7543472 under the allele model, homogenous model and recessive model; genotypes of rs11260867 under the heterogenous model and dominant model were associated with ARC risk. This was the first meta-analysis concerning these three SNPs in ARC risk.

Cataract is the single largest contributor to blindness in the world. Age is the major risk factor for cataract. Even though several measures have been identified as a solution for cataract treatment [35, 36], there is no obvious therapeutic benefits, and established medical treatment for better prevention and treatment of cataract was not built [37]. Moreover, this disease has a strong genetic component. Therefore, understanding of genetic polymorphisms within the lens may provide an insight into the process of cataract onset.

OGG1 is involved in multiple vital processes. Recent studies showed that OGG1 was highly expressed in the embryonic brain, and lack of OGG1 might cause severe brain defects in brain integrity, balance and mobility [38]. The acetylation of OGG1 was shown to play an important etiologic role in regulating its function in response to DNA damage [39], and could be one of the mechanisms for ARC development [40]. Xu et al. proved that OGG1 might increase in lens epithelium cells with ARC, and the alteration of OGG1 level was associated with the location and opaque degrees of lens [41]. Wang et al. proved that the reduced OGG1 expression was correlated with hypermethylation of a CpG island of OGG1 in lens cortex of ARC [42]. In addition, OGG1 mutations might delay the repair of oxidative DNA damage, and be associated with increased disease risk [43]. Ali et al. showed that OGG1 mutation may prove to be a good candidate of better diagnosis, treatment, and prevention of breast cancer [44]. Zhang et al. suggested that OGG1 Ser326Cys polymorphism might be associated with increased risk of ARC [31]. While Su et al. did not find a correlation between OGG1 variants and ARC risk in Han Chinese from the Jiangsu Eye Study [45].

EPHA2 is an epithelial cell tyrosine kinase, and was shown to be enriched in adult tissues [46]. It is highly expressed in aggressive human cancers. During tumor progression, EPHA2 receptor can gain ligand-independent pro-oncogenic functions due to Akt activation and reduced ephrin-A ligand engagement [47]. Moreover, EPHA2 can function as a therapeutic target for antibody therapy of cancers and diseases [48, 49]. Dunne et al. found that EPHA2 was a key driver of invasion and migration and a synthetically lethal target in KRASMT colorectal cancer, indicating that EPHA2 was a poor prognostic marker [50]. Kato et al. showed a promising role for EPHA2 as a target for antibody treatment in melanoma and enhanced the therapeutic effect as an agonistic antibody to EPHA2 [51]. Genetic and pharmacological inhibition of EPHA2 induces apoptosis and abrogates tumorigenic growth of tumor cells [52]. Recent studies have identified EPHA2 as a surprisingly abundant plasma membrane component in cells of the ocular lens [53]. Mutations in EPHA2 have been shown to underlie inherited forms of cataract in humans [54, 55]. Common variants in EPHA2 have been associated with the much more prevalent age-related form of cataract. Dave et al. showed that mutations in EPHA2 accounted for 4.7 % of inherited cataract cases in South-Eastern Australia, and a rare variant rs139787163 was potentially associated with increased susceptibility to cataract, providing a link between congenital and age-related cataract [56]. Furthermore, the cytoprotective and antiapoptotic function of EPHA2 in lens epithelial cells was abolished by the functional polymorphisms [52]. These results indicated the potential role of EPHA2 in maintaining lens clarity during aging by promoting cell viability.

Several limitations were presented in this meta-analysis. First of all, the number of included studies for each SNP was small, future large-scale researches with more ethnicities are needed to further evaluate the relationship. Secondly, between-study heterogeneity was presented in several comparisons. Thirdly, we did not conduct the subgroup analysis by ethnicities due to the less data, which should be concerned in the future. Lastly, gene-gene and gene-environment interactions were not addressed in our meta-analysis.

## Conclusions

Our results indicated that CG genotype and GG+CG genotype of OGG1 rs1052133 might be risk factor for ARC, especially in cortical cataract risk. The T allele and TT genotype of EPHA2 rs7543472; CG genotype and GG+CG genotype of EPHA2 rs11260867 were associated with cortical cataract risk. Future studies are still required to re-evaluate the results of OGG1 and EPHA2 polymorphisms in ARC risk.

## Abbreviations

ARC: Age-related cataract
BER: Base excision repair
CI: Confidence interval
EPHA2: Eph-receptor tyrosine kinase-type A2
HWE: Hardy-Weinberg equilibrium
OGG1: 8-oxoguanine DNA glycosylase-1
OR: Odds ratio
SNP: Single nucleotide polymorphism
